# Isolated aortic root dilation in homocystinuria

**DOI:** 10.1007/s10545-017-0094-7

**Published:** 2017-10-04

**Authors:** Massimiliano Lorenzini, Nishan Guha, James E. Davison, Alex Pitcher, Bejal Pandya, Helena Kemp, Robin Lachmann, Perry M. Elliott, Elaine Murphy

**Affiliations:** 1 University College London Institute for Cardiovascular Science and Barts Heart Centre, St. Bartholomew’s Hospital, London, UK; 2 Cardiology, Department of Experimental, Diagnostic and Specialty Medicine, Alma Mater Studiorum University of Bologna, Bologna, Italy; 3 Department of Clinical Biochemistry, John Radcliffe Hospital and Nuffield Division of Clinical Laboratory Sciences, University of Oxford, Oxford, UK; 4 Metabolic Medicine, Great Ormond Street Hospital for Children NHS Foundation Trust, London, UK; 5 Division of Cardiovascular Medicine, Radcliffe Department of Medicine, University of Oxford, Oxford, UK; 6 Adult Congenital Heart Disease Department, Barts Heart Centre, St. Bartholomew’s Hospital, London, UK; 7 Department of Clinical Biochemistry, North Bristol NHS Trust, Bristol, UK; 8 Charles Dent Metabolic Unit, National Hospital for Neurology and Neurosurgery, Queen Square, London, UK

## Abstract

**Background:**

Vascular complications in homocystinuria have been known for many years, but there have been no reports to date on involvement of the ascending aorta.

**Methods:**

We conducted a cross-sectional study of patients with homocystinuria, known to a single metabolic centre, and evaluated in 2016 with a transthoracic echocardiogram. Aortic root dilation was defined as Z-score ≥ 2.0 SD, and graded mild (Z-score 2.0–3.0), moderate (Z-score 3.01–4.0) and severe (Z-score > 4.0).

**Results:**

The study population included 34 patients, median age of 44.3 years (IQR 33.3–52.2), 50% males, 69% diagnosed aged <18 years and 29% pyridoxine-responsive. Eight (24%) had a history of hypertension. Seven patients (21%) were found to have a dilation of the aortic root, mild in two cases (6%), moderate in four (12%) and severe in one (3%). None had dilation of the ascending aorta. Significant aortic regurgitation, secondary to moderate aortic root dilation, was documented in two patients. A single patient had significant mitral regurgitation due to prolapse of both valve leaflets, as well as mild aortic root dilation. Comparing patients with a dilation of the aortic root to those without, there were no significant clinical, laboratory or echocardiographic differences, with the only exception being that the diameter of the ascending aorta was larger in the group with a dilated aortic root, albeit within normal limits.

**Conclusions:**

A subset of patients with homocystinuria have isolated dilation of the aortic root similar to that observed in Marfan syndrome.

**Electronic supplementary material:**

The online version of this article (10.1007/s10545-017-0094-7) contains supplementary material, which is available to authorized users.

## Introduction

Homocystinuria (HCU, OMIM 236200) is a rare autosomal recessive genetic disease caused by cystathionine β-synthase (CBS, EC 4.2.1.22) deficiency (Mudd 2011). The phenotype is variable and can include: Marfanoid appearance, mental retardation, epilepsy, vascular complications, ectopia lentis and osteoporosis (Mudd et al. 2001). Based mainly on genotype (Mudd et al. 2001), a subset of patients respond to pyridoxine (vitamin B6), these patients tend to present later, and with a milder phenotype (Mudd et al. 1985). Treatment for non-pyridoxine responsive patients includes a combination of a restricted protein diet, methionine-free amino acid supplementation, vitamin B12, folate and betaine.

Thromboembolic disease is a major cause of morbidity and mortality in HCU. In an untreated cohort of HCU patients, 50% experienced a thromboembolic event by the age of 30 and thromboembolism was reported as a significant factor in 80% of deaths (Mudd et al. 1985). Accelerated atherosclerosis is also known to be associated with HCU (Mudd et al. 1985; Yap et al. 2001; McCully 2015). No clinical trials have investigated the issue, but observational studies (Wilcken and Wilcken 1997; Yap et al. 2000, 2001) have described a lower incidence of vascular complications in treated patients compared to the expected rates derived from a historical untreated cohort (Mudd et al. 1985).

Following the incidental documentation of aortic root dilation in a patient with HCU, patients at our centre were systematically screened with an echocardiogram in order to investigate the prevalence of dilation of the ascending aorta in this population.

## Methods

We conducted a cross-sectional study of patients with HCU known to the Charles Dent Metabolic Unit of the National Hospital for Neurology and Neurosurgery, London (*N* = 32), the John Radcliffe Hospital, Oxford (*N* = 1) and Southmead Hospital, Bristol (*N* = 1) who underwent a transthoracic echocardiogram in 2016. The biochemical diagnosis of HCU was based on plasma total homocysteine (Hcy) levels, according to the current recommendations (Morris et al. 2016) and patients were not routinely genotyped. Plasma hypermethioninemia (>45 μmol/L) was documented in 30 of the 34 patients. Cystathionine was not routinely measured. Of the remaining four patients, methionine levels were either ≤45 μmol/L or could not be traced. All four of these patients were clearly documented to be responsive to pyridoxine. As universal newborn screening for HCU only commenced in the UK in 2015, all of these adult patients were either diagnosed following clinical presentation or as part of a family screen following diagnosis in an index sibling. Clinical details were obtained from electronic records. Pyridoxine-responsiveness was determined at diagnosis and was defined as a sustained reduction of plasma total Hcy to <75 μmol/L following treatment with pyridoxine, with normal folate and B12 levels, and without the need for betaine or dietary modification. As part of routine clinical follow up patients also underwent bone mineral densitometry. Reduced bone mineral density was defined as Z- score < −2.0 or T-score < −1.0.

Aortic root dimension was obtained in the parasternal long axis view, measuring the maximal diameter at the sinuses of Valsalva, according to the current recommendations (Lang et al. 2015). The predicted aortic root diameter was calculated considering age, gender and body surface area (BSA, calculated using the Dubois formula), according to Devereux et al. (2012) by using the following formula: 2.423 + (age [years] * 0.009) + (BSA [square meters] * 0.461) + (gender [1 = man, 2 = woman] * 0.267). Z-score was calculated using SD 0.261 cm. Aortic dilation was defined as Z-score ≥ 2.0 SD, and graded mild (Z-score 2.0–3.0), moderate (Z-score 3.01–4.0) and severe (Z-score > 4.0). The ascending aorta, valvular disease and left ventricular ejection fraction were evaluated according to current guidelines (Lancellotti et al. 2013; Lang et al. 2015).

Cumulative Hcy exposure was calculated as follows: (age at diagnosis * Hcy at diagnosis) + [(age at last follow up – age at diagnosis) * mean Hcy during follow up].

### Statistical analysis

Continuous variables are reported as median (interquartile range), categorical values as number (percentage). Continuous variables were compared using Mann-Whitney test and categorical variables compared using Fisher’s exact test. The relationship between cumulative Hcy exposure and Z-score was assessed using Spearman’s rank-order correlation. A *p*-value <0.05 was considered significant. Statistical analyses were carried out using IBM SPSS Statistics for Windows, version 24.0 (IBM Corp., Armonk, N.Y., USA).

## Results

The study population consisted of 34 patients, with a median age of 44.3 years (IQR 33.3–52.2), 50% of them males. Seventy percent of patients had been diagnosed before the age of 18, and 29% were pyridoxine-responsive. Patients who were not pyridoxine-responsive were advised not to consume excessive protein, but as a restricted protein diet is difficult to initiate and sustain in older patients, only four patients followed a formal prescribed low protein diet with specific HCU-appropriate amino acid supplementation. Twenty-seven (79%) had at least one of the known complications of HCU, the most frequent being lens dislocation (present in 62%). Vascular complications were venous in all cases, no patient had a history of arterial thrombosis. Eight patients (24%) had a history of hypertension. Clinical, laboratory and echocardiogram findings of the overall population are reported in Table 1. Seven patients (21%) were found to have a dilation of the aortic root, this was mild in two cases (6%), moderate in four (12%) and severe in one (3%) (Table 2). The ascending aorta was within normal limits in all cases. Significant (≥ moderate) aortic regurgitation, secondary to moderate aortic root dilation, was documented in two patients. Significant mitral regurgitation due to prolapse of both valve leaflets was found in a single patient who also had mild aortic root dilation. All patients were found to have a non-dilated left ventricle with a normal ejection fraction. Against medical advice, the patient with severe aortic root dilation exercised regularly in the gym, focusing mainly on weight lifting and took protein powder supplements with the aim of increasing muscle mass. The concern in this case was that he might also have a second underlying contributing genetic condition. Sequencing of his genomic DNA against a panel of 20 genes associated with familial thoracic aortic aneurysms identified only heterozygous previously described known pathogenic *CBS* mutations (c.[667-14_667-7del(;)1566del]). This gene panel included the *ACTA2, COL3A1, FBN1, FBN2, FLNA, GATA5, MFAP5, MYH11, MYLK, NOTCH1, PRKG1, SKI, SLC2A10, SMAD3, SMAD4, TGFB2, TGFB3, TGFBR1* and *TGFBR2* genes. Of the four patients with moderate aortic root dilation, two lifted weights regularly in the gym and one of the two also took protein powder supplements.

**Table 1:** Clinical, laboratory and echocardiogram findings of the overall population

		Overall population *N* = 34	Non-dilated aortic root *N* = 27	Dilated aortic root *N* = 7	*p*-value
Age, years (IQR)		44.3 (33.3–52.2)	44.4 (33.0–50.6)	40.9 (33.5–57.5)	0.739
Male, n (%)		17 (50%)	12 (44%)	5 (71%)	0.398
Caucasian, n (%)		24 (71%)	19 (70%)	5 (71%)	1.000
Age < 18 years at diagnosis, n (%)		20/29 (69%)	16/22 (73%)	4/7 (57%)	0.642
Pyridoxine-responsive, n (%)		10 (29%)	8 (30%)	2 (29%)	1.000
Total Hcy, μmol/L (IQR)		96.5 (66.4–117.4)	95.7 (60.7–116.7)	98.7 (72.0–119.7)	0.934
Treatment:	Folate	34 (100%)	27 (100%)	7 (100%)	1.000
	B12	33 (97%)	27 (100%)	6 (86%)	0.206
	Pyridoxine	32 (94%)	26 (96%)	6 (86%)	0.374
	Betaine	22 (65%)	18 (67%)	4 (57%)	0.677
	Low protein diet	4 (12%)	3 (11%)	1 (14%)	1.000
Number of complications:	0	7 (21%)	6 (22%)	1 (14%)	
	1	9 (27%)	6 (22%)	3 (43%)	
	2	14 (41%)	12 (44%)	2 (29%)	0.901
	3	3 (9%)	2 (7%)	1 (14%)	
	4	1 (3%)	1 (4%)	0	
Lens dislocation, n (%)		21 (62%)	15 (56%)	6 (86%)	0.210
Venous thrombosis, n (%)		5 (15%)	4 (15%)	1 (14%)	1.000
Intellectual impairment, n (%)		5 (15%)	4 (15%)	1 (14%)	1.000
Epilepsy, n (%)		2 (6%)	2 (7%)	0	1.000
Reduced BMD, n (%)		16/28 (57%)	14/24 (58%)	2/4 (50%)	1.000
Pancreatitis, n (%)		2 (6%)	1 (4%)	1 (14%)	0.374
History of hypertension, n (%)		8 (24%)	6 (22%)	2 (29%)	1.000
Systolic BP, mmHg (IQR)		124 (115–135)	125 (115–135)	122 (116–145)	0.478
Diastolic BP, mmHg (IQR)		80 (75–80)	80 (75–80)	76 (72–88)	0.452
Aortic root, mm (IQR)		33.5 (28.8–39)	32 (28–36)	43 (39–46)	<0.001
Indexed aortic root, mm/m^2^ (IQR)		18.9 (16.8–20.7)	17.2 (15.6–19.3)	21.6 (20.2–23.9)	<0.001
Indexed ascending aorta, mm/m^2^ (IQR)		16.8 (15.3–17.7)	15.6 (15.0–17.2)	18.1 (17.2–20.8)	0.007
Aortic regurgitation >2+, n (%)		2 (6%)	0	2 (29%)	0.370
Mitral regurgitation >2+, n (%)		1 (3%)	0	1 (14.3%)	0.206
Left ventricular EF, % (IQR)		60 (60–64)	60 (60–64)	60 (59.3–67.3)	0.869

The complications considered were: lens dislocation, venous or arterial thrombosis, intellectual impairment, epilepsy, reduced BMD and pancreatitis

*Hcy* homocysteine, *BMD* bone mineral density, *BP* blood pressure, *EF* ejection fraction

**Table 2:** Characteristics of patients with a dilated aortic root

Patient	Age	Pyridoxine responsive	History of hypertension	Blood pressure (mmHg)	Treatment	Aortic root (mm)	Regular (> twice per week) isometric exercise in the gym	Taking a non-prescribed protein supplement
1	38	No	No	120/70	F, B12, P, B	39	No	No
2	51	No	Yes	135/85	F, B12, P, B	39	No	No
3	33	No	No	122/72	F, B12, P	43	Yes	Yes
4	41	No	No	145/90	F, B12, P, B	43	Yes	No
5	63	Yes	Yes	160/80	F, B12, P	46	No	No
6	57	Yes	No	116/76	F, B12, P	41	No	No
7	26	No	No	115/75	F, B, Diet	50	Yes	Yes

*F* folate, *B12* vitamin B12, *P* pyridoxine, *B* betaine, *Diet* prescribed low protein diet

**Fig. 1:**
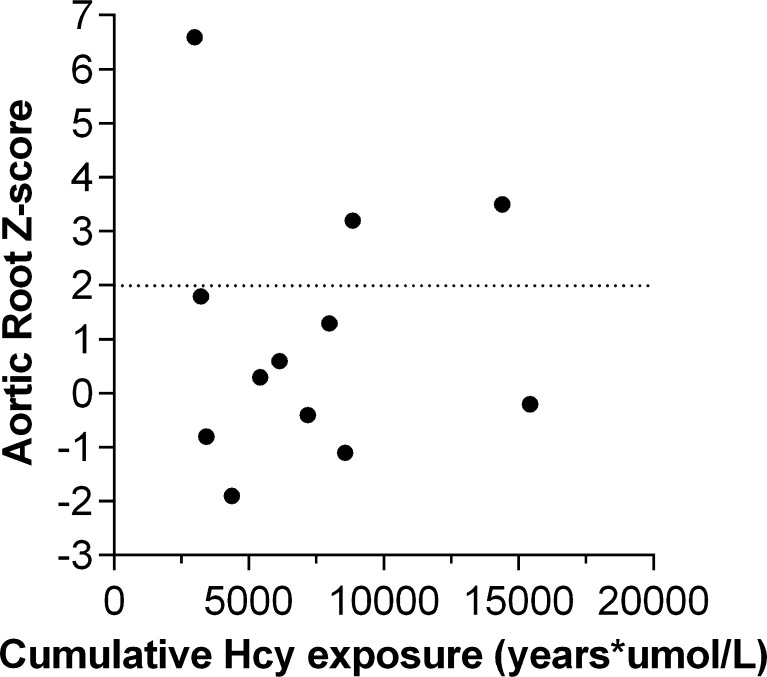
The relationship of aortic root dimensions to lifetime homocysteine exposure. In the subset of patients with available data, no correlation was found between cumulative Hcy exposure [age at diagnosis * Hcy at diagnosis] + [(age at last follow up - age at diagnosis) * mean Hcy during follow up] and aortic root Z-score (*r*_*s*_ − 0.007, *p* = 0.983). A Z-score > 2 indicates aortic root dilation

When comparing patients with dilation of the aortic root to those without, there were no significant clinical, laboratory or echocardiographic differences, with the only exception being that the diameter of the ascending aorta was larger in the group with a dilated aortic root, albeit still within normal limits (Table 1). In the subset of patients with complete available data (*n* = 12), no correlation was found between cumulative Hcy exposure and aortic root Z-score (*r*_*s*_ − 0.007, *p* = 0.983; Fig. 1).

## Discussion

This is the first study to describe the relatively high prevalence of isolated aortic root dilation in HCU, that affects one in five patients, and does not appear to be associated with any of the main demographic, clinical or laboratory findings.

The isolated aortic root dilation we observed in our cohort is similar to that seen in Marfan syndrome (MFS, OMIM 154700), a disease caused in most cases by mutations in the FBN1 gene, that encodes fibrillin-1, but to a lesser degree. By forming a lattice around elastic fibres, fibrillin-1 plays an important structural role in the extracellular matrix. However, haploinsufficiency leading to a weakening of connective tissue does not explain all components of the MFS phenotype, such as long bones (Cañadas et al. 2010). Fibrillin-1 also plays a complex and not completely understood role, interacting with vascular smooth muscle cells and regulating growth factor activity, particularly of TGF-β1. The increased availability of TGF-β1 caused by mutated fibrillin-1 protein leads to matrix metalloproteinase expression and thereby matrix degradation and inflammation (El-Hamamsy and Yacoub 2009). Experimental models have shown that elevated Hcy levels, compatible with those of patients with HCU, can lead to reduction of the disulfide bonds in fibrillin-1 leading to a loss of structure (Hutchinson et al. 2005), and hence to increased TGF-β1 activity (El-Hamamsy and Yacoub 2009). This suggests a pathophysiology partially shared by the two diseases, that would explain the common phenotypic traits, with the lower frequency of aortic disease observed in HCU being due to the different mechanism of fibrillin-1 degeneration, i.e. acquired from exposure to high levels of circulating Hcy rather than from congenital mutation of the protein. The characteristic isolated dilation of the aortic root in MFS is believed to be due to the higher elastic fibre content (containing fibrillin-1) in the aortic root, combined with the wall stress and cyclic torsion to which it is subjected (Cañadas et al. 2010) and it can be speculated that the same principles apply in HCU.

However, while MFS is known to be an important cause of aortic dissection, particularly in young patients, with a prevalence reaching 5% in large dissection registries (Januzzi et al. 2004; Vagnarelli et al. 2015), to the best of our knowledge, to date there have been no reports of aortic dissection in HCU. There have been reports of carotid artery (Kelly et al. 2003; Weiss et al. 2006) or coronary dissection (Granel et al. 2009), as well as two cases of abdominal aortic aneurysm (Yap et al. 2001), but considering the localization of these vascular complications, they possibly had a different, atherosclerotic pathogenesis. Whilst it is possible that the risk of dissection secondary to aortic root dilation is lower in patients with HCU than with MFS, it may be that the lack of reports to date simply reflects the fact that HCU is a rare disease, potentially underdiagnosed when it does not express the classic phenotype, particularly in adults presenting with isolated vascular complications and no other phenotypic features (Brenton 1977; Gaustadnes et al. 2000; Kelly et al. 2003; Linnebank et al. 2003). As aortic root dilation affects only a subset of patients, the absolute number of HCU patients who experience an aortic dissection can be expected to be extremely low. Most patients with dissections secondary to MFS present in their 4th to 5th decade (Januzzi et al. 2004) and so many surviving adult patients with HCU may still be too young to experience this complication. The introduction of newborn screening and earlier treatment aims to lower lifetime Hcy exposure, altering the natural history of the condition and ultimately potentially reducing the risk of aortic root dilation and dissection. To date no studies have reported systematic screening of patients with aortic dissection for HCU, although the *CBS* gene is currently included in various aortopathy gene panels.

The fact that amongst the five patients with moderate or severe aortic root dilation in our series, three regularly lifted weights and two also took protein powder supplements is in line with our pathophysiological hypothesis. Despite the fact that extreme blood pressure values (exceeding 480/350 mmHg) have been recorded by indwelling catheter during weight lifting (MacDougall et al. 1985), the association between isometric exercise and aortic root dilation in athletes remains unclear (Pelliccia et al. 2012). Nevertheless, aortic rupture is a known, albeit rare, cause of sudden death in athletes and was found to be responsible for 2% of cardiovascular deaths in a large US registry (Maron et al. 2009), although it is not known whether these athletes had an undiagnosed aortopathy. Despite the lack of specific outcome data, all current guidelines are extremely restrictive regarding sports participation in patients with MFS or other genetic aortopathies (Pelliccia et al. 2005; Braverman et al. 2015), and based on our findings it is reasonable to advise patients with HCU against strenuous exercise, particularly if isometric.

The fact that no correlation was found between cumulative Hcy exposure and aortic root Z-score does not invalidate our pathophysiological hypothesis. The result can be explained by the small size of the subgroup analysis and possibly by the contribution of weight-lifting to the development of aortic root dilation in this population.

Two of the patients with aortic root dilation in this series had a history of hypertension and, despite the fact that ambulatory blood pressure values were satisfactory, this could be considered a confounder. The prevalence of a history of hypertension however, did not differ significantly between the two groups, and the relationship between hypertension and aortic root dilation, remains controversial despite the large number of studies that have investigated the issue (Mulè et al. 2016). The main determinants of aortic root size are known to be age, gender and body size, all are considered in the Z-score formula proposed by Devereux et al. that was used in this study. Compared to the previous, longstanding nomograms, this formula has been found to perform better in patients with MFS (van Kimmenade et al. 2013), as previous methods did not consider age and gender, as well as being derived from a much smaller cohort (Roman et al. 1989).

Our observations require confirmation in larger, multicentre cohorts. However, considering the improved life expectancy of patients with HCU with contemporary management, that aortic dissection secondary to aortic root dilation is a potentially preventable life-threatening complication, and that dimensions of the proximal aorta can be cheaply and non-invasively assessed with a transthoracic echocardiogram, all patients should be offered screening for aortic root dilation. Losartan inhibits TGF-β mediated activation of extracellular metalloproteinase (Habashi et al. 2006) and is widely used in MFS having been shown to slow aortic root dilation compared to beta-blockers or placebo, even though it does not seem to reduce adverse clinical events (Gao et al. 2016). Based on our pathophysiological hypothesis, Losartan should be strongly considered as treatment in patients with HCU and hypertension, but further evidence is required before it can be routinely recommended in patients with HCU, aortic root dilation and normal blood pressure. Currently, in our unit, those patients with a dilated aortic root at baseline are followed as deemed clinically appropriate by the cardiac team. Those patients with a normal aortic root at baseline are offered a repeat echocardiogram every 3 years.

## Limitations

A limitation of the present study is the absence of a control group and our findings should be confirmed and the possible modifier role of isometric exercise investigated through larger, case-control studies. Our data on the prevalence of aortic root dilation cannot be compared directly with available literature due to the different definitions of ‘aneurysm’. However, the few available studies suggest that it is a rare condition in the general population and the prevalence in our cohort was decidedly higher than could be expected. Amongst 6971 Japanese adults undergoing chest CT for lung cancer or tuberculosis screening the prevalence of dilation of the ascending aorta (defined as mean diameter for age + 3SD) was 0.04% (Itani et al. 2002). In the Heinz Nixdorf Recall study, aortic aneurysms ≥5 cm on chest CT were found in 0.34% of 4129 German subjects (Kälsch et al. 2013).

Despite the fact that 70% of our population was diagnosed in a pediatric context and have undergone medical follow-up ever since, and that there have been no documented cases of aortic dissection amongst the patients followed up at our centre, this cross-sectional study is not able to determine the risk of aortic dissection in HCU and larger prospective studies will be required.

## Electronic supplementary material

ESM 1 (DOCX 23 kb)
