# Supplementary material for: Isolated aortic root dilation in homocystinuria
Source: J Inherit Metab Dis. 2017 Oct 4;41(1):109–15. doi: 10.1007/s10545-017-0094-7 (PMC5786652; doi:10.1007/s10545-017-0094-7)
Supplement: Supplementary file 1 — (DOCX 23 kb) [file 10545_2017_94_MOESM1_ESM.docx]

**SUPPLEMENTAL TABLE**

| Patient | Supporting diagnostic information | Age at follow-up | Pyridoxine responsive | History of hypertension | Treatment | Mean homocysteine*  (umol/L) | Aortic root (mm) |
| --- | --- | --- | --- | --- | --- | --- | --- |
| 1 | LD | 38 | No | No | F, B12, P, B | 125 | 39 |
| 2 | H, LD, M | 51 | No | Yes | F, B12, P, B | 99 | 39 |
| 3 | H, LD, M | 33 | No | No | F, B12, P | 120 | 43 |
| 4 | LD, M | 41 | No | No | F, B12, P, B | 104 | 43 |
| 5 | M, PR | 63 | Yes | Yes | F, B12, P | 72 | 46 |
| 6 | H, LD, PR | 57 | Yes | No | F, B12, P | 15 | 41 |
| 7 | G, LD | 26 | No | No | F, B, Diet | 95 | 50 |
| 8 | H | 34 | No | No | F, B12, P, B | 133 | 27 |
| 9 | LD | 33 | No | No | F, B12, P, B | 54 | 25 |
| 10 | H, LD, M | 46 | No | No | F, B12, B, Diet | 260 | 29 |
| 11 | SS | 43 | No | No | F, B12, P, B | 111 | 28 |
| 12 | LD | 55 | No | No | F, B12, P | 96 | 32 |
| 13 | LD, M | 48 | No | No | F, B12, P, B | 97 | 29 |
| 14 | H, M, PR | 40 | Yes | No | F, B12, P | 19 | 28 |
| 15 | LD, PR | 51 | Yes | No | F, B12, P | 74 | 36 |
| 16 | M, PR | 36 | Yes | No | F, B12, P | 7 | 28 |
| 17 | M | 19 | No | No | F, B12, P, B | 93 | 26 |
| 18 | H, LD, M | 59 | No | Yes | F, B12, P, B | 149 | 34 |
| 19 | PR | 48 | Yes | Yes | F, B12, P | 39 | 34 |
| 20 | H, M | 22 | No | No | F, B12, P, B, Diet | 113 | 28 |
| 21 | H, PR | 70 | No | Yes | F, B12, P, B | 117 | 33 |
| 22 | LD, M | 25 | No | No | F, B12, P, B | 102 | 28 |
| 23 | M, PR | 44 | Yes | No | F, B12, P | 61 | 31 |
| 24 | SS, LD, PR | 61 | Yes | No | F, B12, P | 72 | 32 |
| 25 | LD, M | 48 | No | No | F, B12, P, B | 85 | 32 |
| 26 | H, M | 22 | No | No | F, B12, P, B, Diet | 157 | 30 |
| 27 | H | 46 | No | No | F, B12, P, B | 88 | 37 |
| 28 | H, PR | 34 | Yes | No | F, B12, P | 26 | 31 |
| 29 | H, LD, M | 33 | No | Yes | F, B12, P, B | 310 | 36 |
| 30 | LD, M | 47 | No | Yes | F, B12, P, B | 103 | 39 |
| 31 | G, LD, PR | 59 | Yes | Yes | F, B12, P | 21 | 36 |
| 32 | H, LD, M | 56 | No | No | F, B12, P, B | 169 | 40 |
| 33 | H, LD, M | 23 | No | No | F, B12, P, B | 110 | 36 |
| 34 | LD, M | 44 | No | No | F, B12, P, B | 68 | 40 |

G – genetic confirmation; E – reduced CBS enzyme activity; SS – sibling screen; H – total homocysteine > 150 umol/L on diagnosis; LD – lens dislocation; M – Marfanoid-like body habitus; PR – pyridoxine responsiveness; F – folate; B12 – vitamin B12; P – pyridoxine; B – betaine; Diet – prescribed low protein diet; *– mean of last 3 total homocysteine measurements
